# Geo-additive modelling of malaria in Burundi

**DOI:** 10.1186/1475-2875-10-234

**Published:** 2011-08-11

**Authors:** Hermenegilde Nkurunziza, Albrecht Gebhardt, Jürgen Pilz

**Affiliations:** 1 Department of Mathematics, Institute of Applied Pedagogy, University of Burundi, Burundi; 2 Department of Statistics, University of Klagenfurt, Austria

## Abstract

**Background:**

Malaria is a major public health issue in Burundi in terms of both morbidity and mortality, with around 2.5 million clinical cases and more than 15,000 deaths each year. It is still the single main cause of mortality in pregnant women and children below five years of age. Because of the severe health and economic burden of malaria, there is still a growing need for methods that will help to understand the influencing factors. Several studies/researches have been done on the subject yielding different results as which factors are most responsible for the increase in malaria transmission. This paper considers the modelling of the dependence of malaria cases on spatial determinants and climatic covariates including rainfall, temperature and humidity in Burundi.

**Methods:**

The analysis carried out in this work exploits real monthly data collected in the area of Burundi over 12 years (1996-2007). Semi-parametric regression models are used. The spatial analysis is based on a geo-additive model using provinces as the geographic units of study. The spatial effect is split into structured (correlated) and unstructured (uncorrelated) components. Inference is fully Bayesian and uses Markov chain Monte Carlo techniques. The effects of the continuous covariates are modelled by cubic p-splines with 20 equidistant knots and second order random walk penalty. For the spatially correlated effect, Markov random field prior is chosen. The spatially uncorrelated effects are assumed to be i.i.d. Gaussian. The effects of climatic covariates and the effects of other spatial determinants are estimated simultaneously in a unified regression framework.

**Results:**

The results obtained from the proposed model suggest that although malaria incidence in a given month is strongly positively associated with the minimum temperature of the previous months, regional patterns of malaria that are related to factors other than climatic variables have been identified, without being able to explain them.

**Conclusions:**

In this paper, semiparametric models are used to model the effects of both climatic covariates and spatial effects on malaria distribution in Burundi. The results obtained from the proposed models suggest a strong positive association between malaria incidence in a given month and the minimum temperature of the previous month. From the spatial effects, important spatial patterns of malaria that are related to factors other than climatic variables are identified. Potential explanations (factors) could be related to socio-economic conditions, food shortage, limited access to health care service, precarious housing, promiscuity, poor hygienic conditions, limited access to drinking water, land use (rice paddies for example), displacement of the population (due to armed conflicts).

## Background

In Burundi, malaria is a major public health issue in terms of both morbidity and mortality with around 2.5 million clinical cases and more than 15,000 deaths each year. In 2001, Burundi was the world's most affected country by malaria [1]. Malaria is the main cause of mortality among pregnant women and children under five years of age, accounting for more than 50% of all cases.

Many studies have been undertaken to understand factors that are associated with malaria in many countries. Most of them found a strong association between malaria and climate [2-5]. For example, the results in [2] suggest that the variability of the climate played an important role in initiating epidemics of malaria in the highlands of East Africa. A significant positive correlation between the number of malaria cases and temperature and rainfall has been identified. Pemola and Jauhari [3] found higher positive correlation between monthly malaria parasite incidence and climatic variables (temperature, rainfall and humidity) in Dehradun, India. Gallup and Sachs [4] suggested that the location and severity of malaria are mostly determined by climate and ecology. Bouma *et al *[5] concluded that rainfall and humidity were able to predict malaria rates fairly well in Pakistan.

However, other studies on the same topic suggested that factors other than climate may explain the distribution of malaria [6-11]. For example, Cox *et al *[6] noted that the relatively high rates of malaria morbidity in Africa could result from poor access to health services, inadequate case management, overwhelmed health services, poor immunological competence because of malnutrition, a general disruption to livelihoods because of often-associated flooding, or a combination of these factors. Patz and Lindsay [7] suggested the existence of many variables affecting malaria transmission beside the climatic changes, such as environmental factors, the population growth, a limited access to health care systems, and lack of or unsuccessful malaria control measures. Kigbafori *et al *[8] concluded that risk factors for malaria infection include age, socioeconomic factors, not sleeping under a bed net, lack of health care facilities and various environmental features, such as vegetation, rainfall and distance to rivers. Tren [9] suggested that though climate can affect the incidence of malaria, man's economic activities and malaria control policy play a very important role in the incidence of the disease. Hay *et al *[11] suggested that the claimed association between local malaria resurgence and regional changes in climate, in Eastern Africa, is overly simplistic. They suggest that economic, social and political factors explain recent resurgence in malaria and other mosquito-born diseases with no need to invoke climate change.

In this study, a geo-additive model is proposed to understand the dependence of malaria cases on spatial effects and climatic covariates including rainfall, maximum and minimum temperature, maximum and minimum humidity in Burundi.

## Methods

### Study area

Burundi is located in East-central Africa, between 2°20 and 4°27 of latitude south and between 28°50 and 30°53 of longitude east; the altitude varies between 775 metres (Lake Tanganyika) and 2,670 metres (Crest Congo - Nil). Burundi has in general a tropical highland climate with a significant daily temperature variation in many areas [12]. Temperature also varies significantly from one region to another mainly due to differences in altitude. The area in the central plateau is cool, with temperature averaging 20°C. The area near Lake Tanganyika is warmer, averaging 23°C; the areas in the highest mountains are cooler with temperature averaging 16°C. Rain is irregular and falls most heavily in the northwest region [12]. Dry season varies in length with sometimes longer periods of drought. Most parts of Burundi receive rainfall between 130 cm and 160 cm per year [12]. Bounded on the north by Rwanda, in south-east by Tanzania and in west by the Democratic Republic of Congo, Burundi covers an area of 27,834 km^2 ^(of which 2,634 km^2 ^are occupied by Tanganyika Lake) and has a population estimated at about 8 million. In terms of habitat, it remains essentially rural, with 91.6% of the population living in rural area. The urban population is 8.4% with an annual growth rate of 5.7%. The Burundi population is young: 46.1% are under 15 years of age, while people aged 60 and above represent only 5.4%. With an average density of 266 inhabitants per km^2^, a population growth rate of 3.44% and a total fertility rate of 6 children per woman, Burundi is one of Africa's most densely populated countries [13]. Burundi is structured in 17 provinces. The epidemiological profile can be summarized as follows. The health system suffers from a shortage of qualified personnel with 1 doctor per 34,750 inhabitants and 1 nurse for 3,500 inhabitants [13]. 17.4% of patients do not have access to health care, while 81.5% of patients are forced to go into debt or sell property to pay the health costs. There is a big disparity between the capital Bujumbura and the remainder of the country as 80% of doctors and more than 50% of nurses are engaged in Bujumbura. Responsible for more than 50% of hospital deaths in children under five years of age and more than 40% of all consultations in health centres, malaria is undoubtedly the main public health problem, the main cause of mortality and morbidity in Burundi [13].

### Data description

The goal in our study is to understand the dependence of malaria cases on factors such as climatic variables and spatial (correlated and uncorrelated) effects in Burundi. Monthly data on malaria morbidity in Burundi over 12 years (from 1996 to 2007) were collected from EPISTAT (Epidemiology and Statistics in Burundi) [14], a department of the Burundi Ministry of health in charge of collecting and storing data on epidemiology all over the country. The well-known nearest neighbour method was used to fill the missing data (~5%). The estimated population for each province, for the study period, was obtained from the Institute of Statistics and Economic Studies in Burundi (ISTEEBU)[[15] Malaria incidence in a given province was computed by dividing the number of malaria cases by the total population of the province, assuming that the whole population is susceptible. Monthly data on cumulative precipitation, monthly average of daily maximum temperature, minimum temperature, maximum humidity and minimum humidity for 1996-2007 was obtained from the Geographic Institute of Burundi (IGEBU) [16]. The record of these variables from 1996 through 2007 has remained uniform, with the same calibration and the same precision. The missing data (2% - 3%) were filled by the same method as in Malaria data (nearest neighbour and cross-validation). Data for three provinces (Bubanza, Bujumbura rural and Cibitoke) were not available for the study period; they were estimated using ordinary kriging [17]. The data are available on different scales and units (malaria incidence and humidity are unit free, rainfall is measured in centimetre (cm), temperature in degree centigrade (°C)). They were then standardized to avoid the effect of scale in the modelling.

### Model formulation

In a previous study [18], assuming that climatic covariates have a nonlinear effect on malaria incidence and based on the Akaike information criterion (AIC) using the algorithm described in [23], the following generalized additive mixed model (GAMM) [24] was proposed to assess the dependence of malaria cases on climatic variables.(1)

Here *η_it _*is the predictor of malaria incidence assumed to have a gamma distribution, *R_nit _*is the rainfall, *H_xit _*is the maximum humidity, *T_xit _*is the maximum temperature and *T_nit _*is the minimum temperature, of the province *i *in month *t*. *T_xp _*,*T_np _*,*H_xp _*are the same variables for the previous month. *f*_1_, ···, *f*_4 _are unknown nonlinear smooth functions of the covariates. The *α_i _*(*i *= 1,···, 3) are the regression coefficient of the linear effects. *α*_0 _is the intercept (accounting for unmeasured covariates).*ε_it _*is the error.

The aim here was to assess the climatic factors that are highly associated with monthly malaria incidence in Burundi; hence spatial effect was not included in the model. The results have shown that malaria incidence in a given month is positively associated with the minimum temperature in the previous month. In this study, the GAMM in (1) is replaced by a geo-additive model by incorporating the spatial effects as follows [25-32].(2)

Here, as above, *f*_1_,···, *f*_4 _are nonlinear smooth functions of the metrical continuous covariates and *f_spat _*is the effect of the spatial covariate *p_i _*,(*i *= 1, ···, 17) representing province *i*. The spatial effect *f_spat _*is then split up into correlated (structured) and uncorrelated (unstructured or random) effects as follows [30,31].(3)

The logic behind this is that a spatial effect is usually a combination of many unobserved influences, some of them obeying a strong spatial structure and others being present only locally [26-31,33]. Eq. (2) is then written as(4)

This geo-additive model assumes that the nonlinear effects *f*_1_,···, *f*_4 _are the same for all provinces.

### Prior assumptions and inference

For Bayesian inference, the unknown functions *f*_1_,...., *f*_4 _in predictor (4), the vector of the linear effects parameter *α *= (*α*_0_, *α*_1_, *α*_2_, *α*_3_), are considered as random variables and are supplemented by prior assumptions. In the absence of any prior knowledge, diffuse priors are the appropriate choice for fixed effects parameters, i.e. *p*(*α_i _*) ∝ *const *[32,34,35]. Another common choice are highly dispersed Gaussian priors [31].

For the continuous (smooth) functions *f*_1_,...., *f*_4 _, a second order random walk prior is considered for *f *defined as follows. Consider the case of a metrical covariate *x *with equally spaced observations *x_i _*, *i *= 1, ···, *m *, *m *≤ *n *(*n *is the number of observations). Suppose that *x*_(1) _< ··· <*x*_(*t*) _< ··· <*x*_(*m*) _is an ordered sequence of distinct values for a covariate and define *f*(*t*) = *f*(*x*_(*t*)_). The second order random walk is then defined by(5)

with Gaussian errors *u*(*t*) ~ *N*(0, *τ*^2^) and diffuse priors *f*(1) ∝ *C^st ^*and *f*(2) ∝ *C^st^*, for initial values. A second order random walk penalizes deviations from the linear trend 2*f*(*t*-1)-*f*(*t*-2) [33,36,37]. For the spatially correlated effect *f_str _*, Markov random field prior is chosen [32,38]. This prior indicates spatial neighborhood relationship. For geographical data, a common assumption is that two sites or regions *r*_1 _and *r*_2 _are neighbors if they have a common boundary [25-32]. Thus, a spatial extension of the random walk model leads to the following conditional spatially autoregressive specification [25-32](6)

Here *N_s _*is the number of adjacent provinces and *p' *∈*p *denotes that province *p' *is a neighbour of province *p*. The prior is called a Markov random field (MRF) [31,32,38]. We define provinces as neighbours if they share the same boundary and assume that the effect of a province *p *is conditionally Gaussian with expectation equals to the mean of the effects of neighbouring provinces and a variance that is inversely proportional to the number of its neighbours *N_s _*[26,31]. The conditional mean of *f_str _*(*p*) is an unweighted average of function evaluations of neighbouring provinces. For the spatially uncorrelatated (unstructured) effect, *f_unstr _*are assumed to be i.i.d. Gaussian (this is a common assumptions) [26-31]:(7)

The variance parameters  control the trade-off between flexibility and smoothness [36,37]. They are also considered as unknown and estimated simultaneously with corresponding unknown functions *f_j _*. Weakly informative inverse Gamma hyperprior  are assigned to . The corresponding probability density function is given by [39].(8)

Using proper priors for  (*a_j _*> 0 and *b_j _*> 0) ensures propriety of the joint posterior [39].

Bayesian inference is based on the posterior of the model and is carried out using MCMC simulation techniques. For the predictor (4), let *γ *denotes the vector of all unknown parameters in the model. Then, under conditional independence assumptions, the posterior of the model is given by [26-31].(9)

The full conditionals for the parameter vectors *f_j _*, *j *= 1, ···. 4 as well as the full conditionals for *f_str _*, *f_unstr _*are multivariate Gaussian. The MCMC simulation is used for successive draw of  from the full conditionals [26-31]. The model is implemented in BayesX, a public domain software for Bayesian inference in structured Additive Regression Models [40]. Only the main effects are modelled. The effects of two-factor interactions are assumed to be smaller and are omitted. The main reason is that we wish to preserve the simplicity and easy interpretation of the effects, which are often lost by including interactions [24]. The effects of the continuous covariates are modelled by cubic p-splines [41,42] with 20 equidistant knots and second order random walk penalty [36,43]. Positive hyperparameters *a *= 0.0001 and *b *= 0.0005 have been chosen for *τ*^2 ^to ensure the propriety of the posterior [39]. 12,000 iterations of the MCMC were run with a burn-in phase of 2,000 iterations. Thinning was applied to the Markov Chain to reduce autocorrelations, by requiring the programme to store only every 10^th ^sampled parameter. Single block updating scheme is adopted, with inverse weighted least square (IWLS) proposal [35,37]. Sensitivity of the results with respect to changes in the hyperparameters *a *and *b *was checked. The model was then re-estimated with different choices for the hyperparameters *a *and *b *for each effect in the model by (*a *= 1, *b *= 0.005); (1 = 0.001, *b *= 0.001); (*a *= 0.001, *b *= 0.005); (*a *= 0.001, *b *= 0.005) (*a *= 0.0001, *b *= 0.0001); (*a *= 0.001, *b *= 0.0005) to assess the dependence of results on minor changes in the model assumptions. The results showed any significant change.

## Results and discussion

The aim in this study is to analyse the dependence of malaria cases on factors, such as climatic variables and spatial (correlated and uncorrelated) effects in Burundi. Table 1 presents the estimate of the linear effects parameters.

**Table 1:** Estimate of the linear effects parameters of model (4).

Parameter	Mean	**Std. Dev**.	Median	95% Credible Interval(CI)
*α*_0_	0.8470	0.0551	0.8482	[0.7410, 0.9586]
*α*_1_	-0.0303	0.0134	-0.0300	[-0.0563, -0.0019]
*α*_2_	0.0144	0.0156	0.0140	[-0.0152, 0.0463]
*α*_3_	0.0595	0.0142	0.0591	[0.0323, 0.0873]

**Figure 1:**
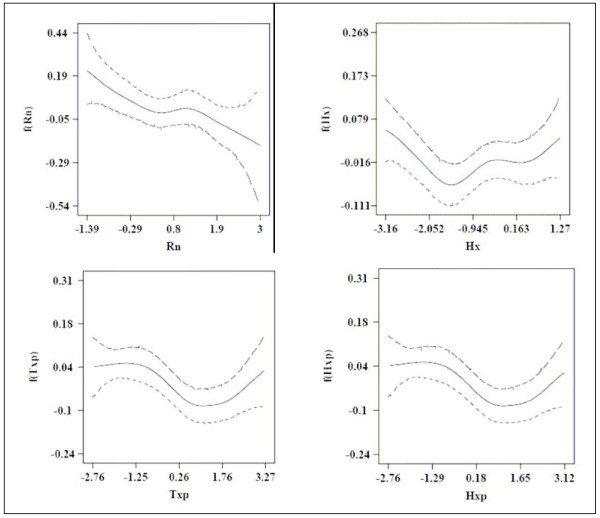
**Nonlinear effect of the continuous covariates, with 95% credible interval**.

In Table 1. *α*_0_, *α*_2 _and *α*_3 _have a positive mean. *α*_0 _and *α*_3 _have a positive credible interval (CI). *α*_1 _has a negative mean with a negative 95% credible interval (CI). These results suggest that malaria incidence in a given month is positively associated with the minimum temperature of the same month and more strongly with the minimum temperature of the previous month. In contrast, the results suggest that malaria incidence in a given month is negatively associated with maximum temperature of the same month. *α*_0 _(the intercept) has the largest value, suggesting that unmeasured covariates have larger effect on malaria incidence. Figure 1 presents the nonlinear effects in model (4), with 95% credible interval. The upper-left plot of Figure 1 suggests that malaria incidence in a given month is negatively associated with rainfall of the same month. The above results may be explained as follows. Minimum temperature is the most influential factor of malaria incidence as it is observed at night and mosquitoes are active only at night; by day time they hide themselves in houses or vegetation. Moreover, when the night temperature is high, people do not cover themselves, increasing the risk of being bitten by Mosquitoes. Furthermore, due to the development cycle of the parasite into mosquitoes and the incubation period, those who became ill in a given month were bitten by mosquitoes in the previous month. This explains why malaria incidence in a given month is strongly associated with the minimum temperature of the previous month.

**Figure 2:**
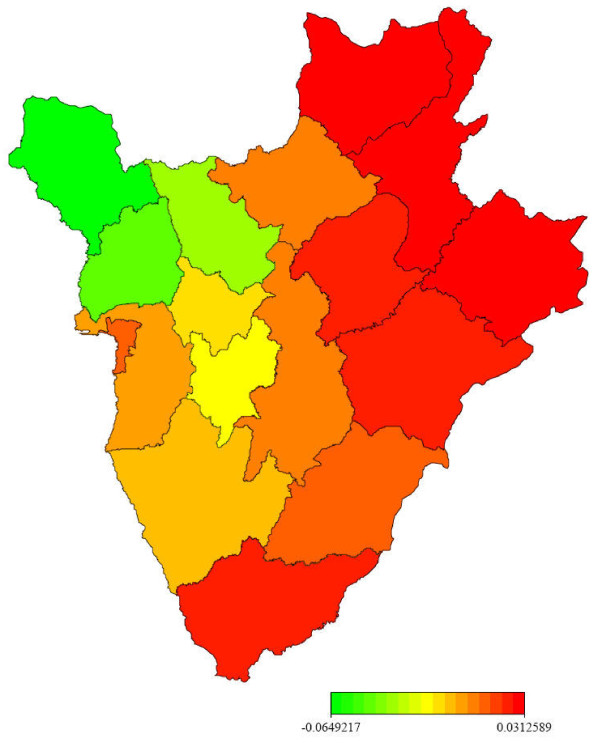
**Posterior mean estimate of the structured spatial effect**.

**Figure 3:**
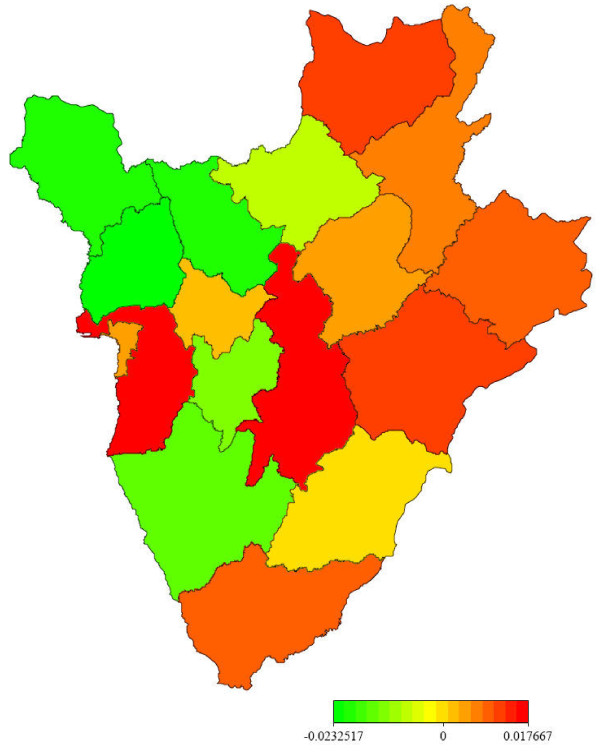
**Posterior mean estimate of the random spatial effect**.

In contrast, the maximum temperature has a negative effect because mosquito's development is interrupted at higher temperature [44]. Too much rainfall may flush away the breeding larvae, decreasing the number of mosquitoes. Figures 2 and 3 show distinct spatial patterns that point to the influence of variables other than climate on malaria.

Figure 2 presents the posterior mean estimates of the structured smooth spatial component *f_str _*. The map shows two main patterns: the western part, less affected by structured effect and the eastern part displaying a high risk of structured spatial effect. Figure 3 displays the posterior mean estimates of the unstructured (random) component *f_unstr _*. The map shows similar trend as in Figure 2, but two provinces (Bujumbura Rural and Gitega) seem to present higher risk than others. This is probably because those provinces have a high population density, but more explanations are needed to understand the clear difference among provinces. The generated maps in this study could be used for targeting provinces of high risk of malaria in view to initiate control policy.

## Conclusion

In this paper, semiparametric models were used to model the effects of both climatic covariates and spatial effects on malaria distribution in Burundi. The spatial analysis was based on a geo-additive model in which the province is the geographic unit of analysis. The spatial effect was split into smooth structured and unstructured (random) components. Inference was fully Bayesian and was based on Markov chain Monte Carlo techniques. The effects of climatic covariates and the effects of other spatial determinants were estimated simultaneously, in a unified regression framework. The obtained results suggest that malaria incidence in a given month is positively associated with the minimum temperature of the same and the previous months. In contrast, it is found that malaria incidence is negatively associated with rainfall and maximum temperature of the same month. From the spatial effects, important spatial patterns of malaria that are related to factors other than climatic variables were identified without being able to explain them. Potential explanations (factors) could be related to socio-economic conditions, food shortage, limited access to health care service, precarious housing, promiscuity, poor hygienic conditions, limited access to drinking water, land use (rice paddies for example), displaced population camps (due to armed conflicts) [6,10]. Unfortunately most of these factors are difficult to quantify in the context of poor countries like Burundi, where the record of such features is rare or nonexistent.

## Competing interests

The authors declare that they have no competing interests.

## Authors' contributions

HN collected the data, performed the statistical analysis and drafted the manuscript. AG and JP reviewed and improved the manuscript. All authors read and approved the final manuscript.
